# Refractory circulatory shock following intentional polypharmacy antihypertensive and NSAID overdose: Clinical recovery temporally associated with sequential intravenous lipid emulsion and methylene blue — A case report

**DOI:** 10.1016/j.toxrep.2026.102371

**Published:** 2026-11-06

**Authors:** Syed Hussain Danial, David Michael Honan

**Affiliations:** a Department of Anaesthesia, Wexford General Hospital, HSE Dublin & South East, Newtown Road, Carricklawn, Wexford Town, Wexford Y35 Y17D, Ireland; b School of Medicine, University College Dublin, Belfield, Dublin 4, Ireland

**Keywords:** Calcium channel blocker poisoning, Circulatory shock, Fat emulsion, Methylene blue, High-dose insulin, Acute kidney injury

## Abstract

**Background:**

Calcium channel blocker (CCB) overdose carries substantial mortality without advanced organ support. Ingestion of a fixed-dose antihypertensive combination with a non-steroidal anti-inflammatory drug (NSAID) is an uncommon polypharmacy toxidrome. The role of intravenous lipid emulsion (ILE) and methylene blue in refractory cases remains poorly defined.

**Case report:**

A 64-year-old man (135 kg) ingested approximately 300 mg amlodipine, 300 mg perindopril, 75 mg indapamide and 15,000 mg naproxen with ethanol. Despite apparent stability on arrival he deteriorated to 77/47 mmHg, pH 7.20 and lactate 10.5 mmol/L. Seven hours of escalating treatment — noradrenaline (peak 58 mcg/min), vasopressin, calcium and high-dose insulin euglycaemic therapy (HIET) — failed to restore haemodynamic targets. Intralipid 20% was given (total approximately 8.9 mL/kg), followed 35 min later by methylene blue 140 mg (1.0 mg/kg). Lactate fell from 10.5 to 6.1 mmol/L over the subsequent 55 min with the noradrenaline dose unchanged. All vasopressors were stopped within 14 h. HIET was accompanied by concurrent glucose, potassium and phosphate nadirs (1.7, 1.7 and 0.27 mmol/L). KDIGO stage 2 acute kidney injury (peak creatinine 303 µmol/L) required no renal replacement therapy and resolved completely (creatinine 81 µmol/L at 3.5 months). The patient was discharged on day 9.

**Conclusion:**

Sequential ILE and methylene blue were temporally associated with rapid metabolic recovery in refractory circulatory shock following polypharmacy antihypertensive overdose. Individual contributions cannot be determined from a single case, and a delayed effect of concurrent HIET cannot be excluded. These agents may represent rescue options when conventional therapy is insufficient.

## Highlights

• Fixed-dose antihypertensive combination with NSAID caused refractory vasoplegic shock.
• Serum lactate fell 42% in 55 min after sequential ILE and methylene blue.
• All vasopressors discontinued within 14 h without VA-ECMO.
• HIET produced simultaneous glucose, potassium, and phosphate nadirs concurrently.
• KDIGO Stage 3 acute kidney injury resolved without renal replacement therapy.

## Graphical Abstract

**Figure:**
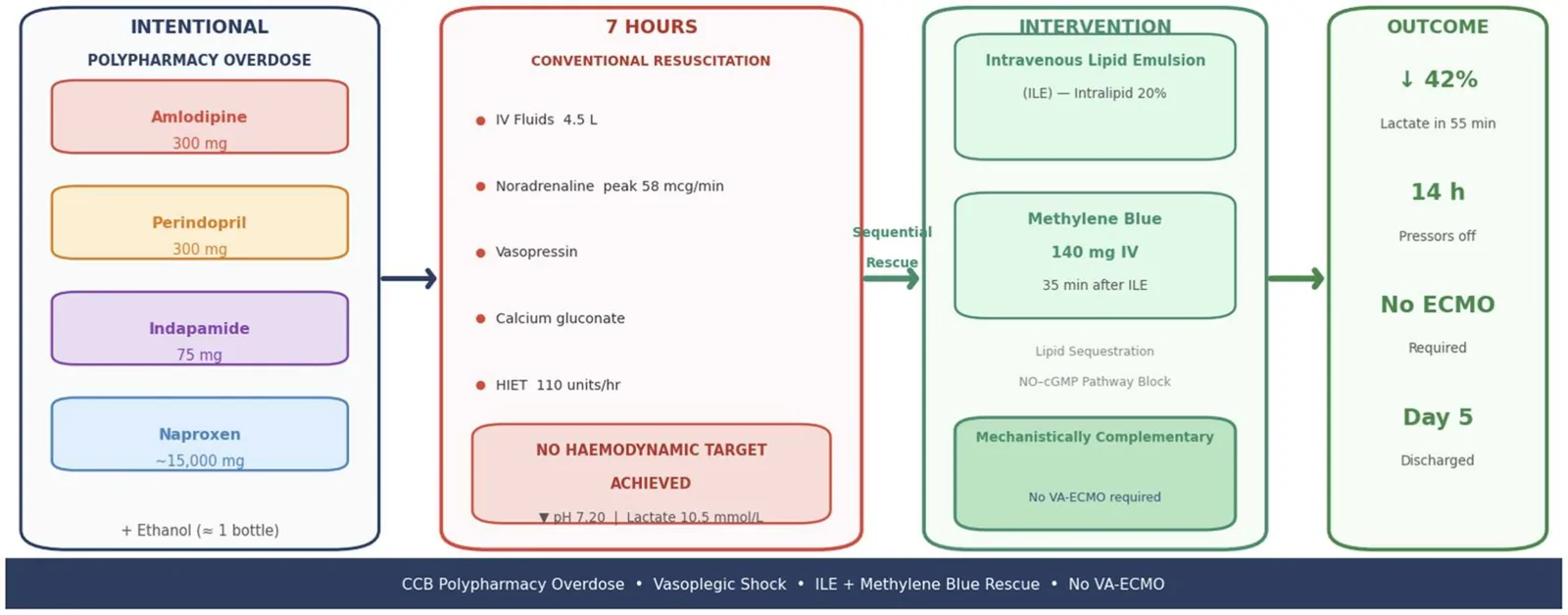

## 1 Introduction

Calcium channel blockers (CCBs) are among the most hazardous cardiovascular drugs in overdose and are associated with substantial reported mortality in the absence of advanced organ support [1]. Fixed-dose antihypertensive combinations concentrate this risk: a single packet of Coverdine (amlodipine 10 mg, indapamide 2.5 mg, perindopril 10 mg) delivers 300 mg of amlodipine together with an ACE inhibitor and a thiazide-like diuretic. When an NSAID is co-ingested, prostaglandin-mediated afferent arteriolar vasodilation, angiotensin II-mediated efferent vasoconstriction and intravascular volume are compromised simultaneously, increasing the risk of acute kidney injury (AKI) [2]. Mengistie et al. recently described massive amlodipine ingestion with an NSAID complicated by multiorgan failure and cardiac arrest, illustrating the severity of this drug combination [3]. Deliberate ingestion of all four drug classes together, however, has rarely been reported.

The systematic review by Cave and Harvey identified animal and limited human data supporting intravenous lipid emulsion (ILE) in lipophilic cardiotoxin poisoning [4], and Levin et al. reported reduced mortality with methylene blue in post-cardiac-surgery vasoplegia [5]. The sequential use of both agents in severe CCB-predominant polypharmacy overdose has not previously been described with detailed temporal physiological data. We report such a case managed in a regional general hospital without on-site veno-arterial extracorporeal membrane oxygenation (VA-ECMO).

## 2 Case report

A 64-year-old man (135 kg) with treated hypertension (Coverdine: amlodipine 10 mg, indapamide 2.5 mg, perindopril 10 mg daily) and a history of depression was found vomiting at home. Antidepressant therapy had been discontinued approximately four years earlier and had not been restarted; the specific agent could not be recalled by the patient and was not documented. He had ingested approximately 30 tablets each of Coverdine (approximately 300 mg amlodipine, 75 mg indapamide, 300 mg perindopril) and naproxen 500 mg/esomeprazole 20 mg (approximately 15,000 mg naproxen) the previous evening, together with approximately one bottle of spirits. Mental health review subsequently characterised the act as impulsive, in the context of recent bereavement and alcohol intoxication. Written informed consent for publication was obtained.

An emergency toxicology screen comprising serum paracetamol and a urine immunoassay for opiates, benzodiazepines, cannabis, tricyclic antidepressants and cocaine was negative. These assays do not detect amlodipine, perindopril, indapamide or naproxen, and the result therefore neither confirms nor excludes the ingested agents; it was used only to exclude the specific co-ingestants tested. Quantitative plasma concentrations of the ingested drugs were not available at our laboratory. Thyroid function was normal and blood and urine cultures were sterile. Blood ethanol was not measured; ethanol co-ingestion is based on the history and the finding of hypothermia alone. The clinical presentation was considered highly compatible with, though not diagnostic of, severe CCB toxicity: hyperglycaemia of 14.0 mmol/L in the context of shock, a heart rate of 64 beats/min despite a mean arterial pressure below 65 mmHg, first-degree atrioventricular block, and a corrected QT interval of 487 ms. TOXBASE guidance was followed throughout.

Prehospital blood pressures were 86/50, 65/41 and 67/26 mmHg with a heart rate of 68–80 beats/min and Glasgow Coma Scale 15. On arrival in the Emergency Department the blood pressure was 113/96 mmHg with a temperature of 33.8°C. The narrow pulse pressure of 17 mmHg was consistent with intense peripheral vasoconstriction secondary to hypothermia, temporarily offsetting the underlying vasodilatory state. The first arterial blood gas showed pH 7.42, lactate 8.5 mmol/L, potassium 2.5 mmol/L, magnesium 0.68 mmol/L, phosphate 0.59 mmol/L and ionised calcium 1.07 mmol/L. Point-of-care B-type natriuretic peptide was 25 pg/mL and troponin was negative. Echocardiography was not performed at any point during the admission; in its absence the shock state is described as refractory circulatory shock with predominantly vasodilatory features, and a contribution from myocardial depression cannot be excluded.

By 08:54 the blood pressure had fallen to 77/47 mmHg and noradrenaline was commenced. Treatment over the following seven hours comprised intravenous fluid resuscitation (4.5 L), noradrenaline escalated to quad-strength concentration (peak 58 mcg/min), vasopressin 0.04 units/min from 12:40, calcium gluconate 10% 30 mL at 10:00, and HIET from 14:00 (actrapid 110-unit bolus then 110 units/h, dosed to an adjusted body weight ceiling of 110 kg in accordance with local practice in obesity) with 10% dextrose. Glucose and potassium were monitored by arterial blood gas at approximately one- to two-hourly intervals throughout (Table 1), targeting glucose 4–8 mmol/L and potassium 3.5–4.5 mmol/L. On transfer to intensive care at 15:10 the blood pressure remained 96/42 mmHg on dual vasopressors, with pH 7.20, lactate 10.5 mmol/L and phosphate 0.27 mmol/L (Table 1; Fig. 1).

**Table 1:** Serial arterial blood gas results and clinical events from Emergency Department presentation to day 2. ᵃ The lactate value of 6.1 mmol/L at 17:00 represents a 42% reduction from the preceding measurement of 10.5 mmol/L at 15:13, recorded while the noradrenaline infusion rate was unchanged. High-dose insulin euglycaemic therapy had been running for approximately three hours at this point and a delayed contribution cannot be excluded. AG, anion gap; BE, base excess; HIET, high-dose insulin euglycaemic therapy; ILE, intravenous lipid emulsion; MAP, mean arterial pressure.

**Time**	**pH**	**Lactate (mmol/L)**	**K⁺ (mmol/L)**	**Glucose (mmol/L)**	**Clinical event / intervention**
08:25	7.42	8.5	2.5	14.0	First ABG; Mg²⁺ 0.68; PO₄ 0.59; iCa²⁺ 1.07; AG 16; ECG 1° AV block, QTc 487 ms
09:07	7.35	9.2	2.6	12.5	Arterial line sited
10:27	7.28	9.2	2.3	10.0	BE −13.2; AG 24; calcium gluconate given 10:00
12:29	7.33	9.6	2.8	9.5	Vasopressin added 12:40
14:13	7.33	9.3	3.4	12.1	HIET commenced 14:00 (110 units/h)
15:13	7.20	10.5	—	9.2	ICU admission; metabolic nadir; PO₄ 0.27; ILE commenced 15:30
16:05	—	—	—	—	Methylene blue 140 mg IV (1.0 mg/kg)
17:00	7.25	6.1	3.1	6.4	First ABG after methylene blue; noradrenaline dose unchangedᵃ
18:50	7.28	4.8	3.06	6.8	Fourth KCl infusion commenced
20:00	7.35	3.7	4.0	6.1	MAP > 65 mmHg; potassium phosphate and MgSO₄ 2 g given
22:30	7.35	3.1	4.2	5.5	Stable
06:00 Day 2	—	—	—	—	All vasopressors discontinued; MAP 80 mmHg
08:39 Day 2	7.36	1.1	4.4	3.9	Hypoglycaemia treated with 50% dextrose 20 mL

**Fig. 1:**
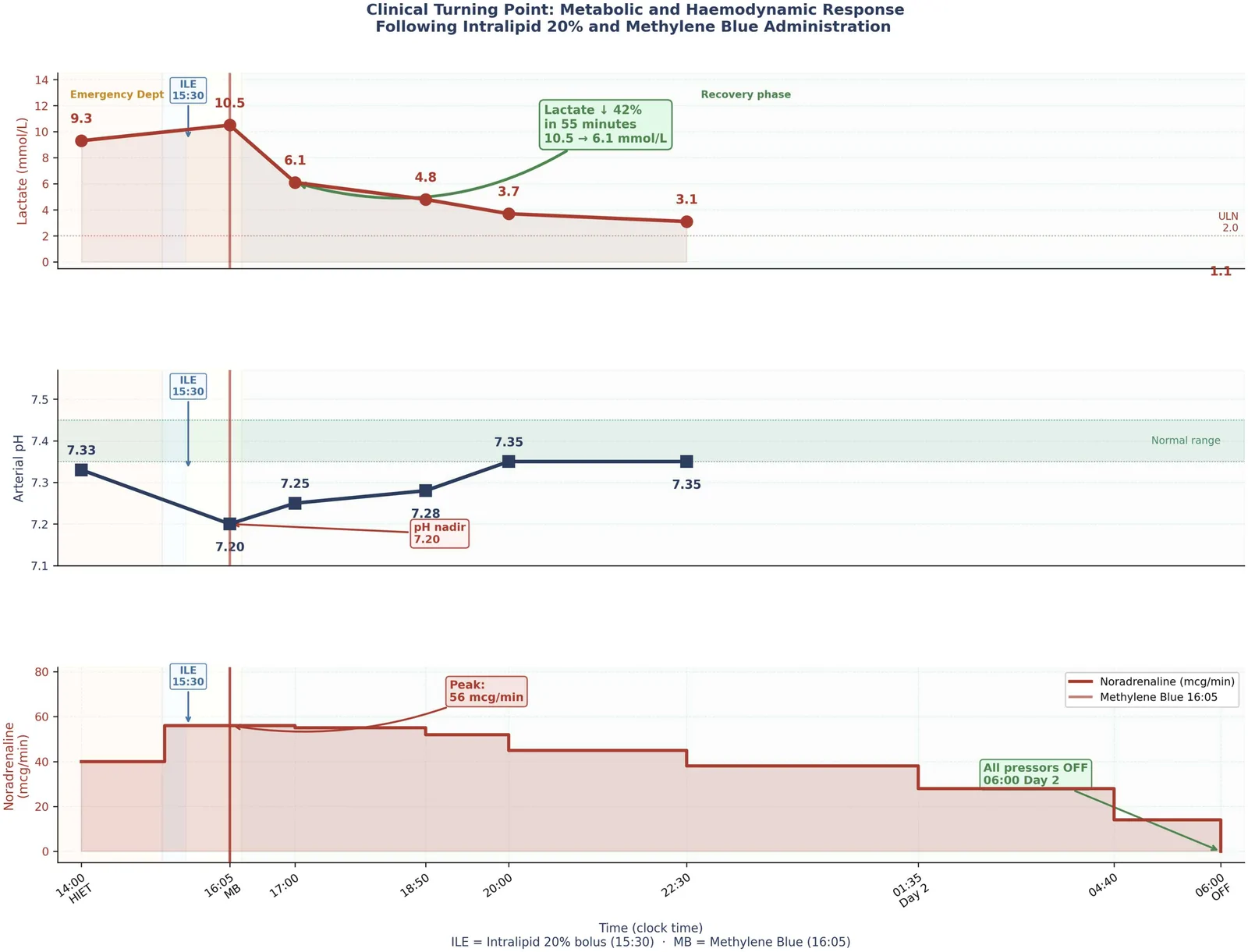
Serial lactate, arterial pH and noradrenaline dose from initiation of high-dose insulin euglycaemic therapy (14:00) to discontinuation of vasopressors (06:00, day 2). Upper panel: serum lactate, showing a 42% reduction from 10.5 to 6.1 mmol/L over 55 min following methylene blue administration at 16:05 (red vertical line). Middle panel: arterial pH. Lower panel: noradrenaline dose, unchanged between 15:10 and 17:00. The temporal relationship is shown without implying causation; HIET had been running for approximately three hours and multiple other therapies were continuing concurrently. ILE, intravenous lipid emulsion bolus commenced 15:30 (blue annotation).

Intralipid 20% was commenced at 15:30, given as a 200 mL bolus over 30 min (1.5 mL/kg) followed by a 1000 mL infusion from 16:00 (7.4 mL/kg), a total of approximately 8.9 mL/kg or 1.8 g/kg. This is within the cumulative ceiling of 12 mL/kg recommended in lipid rescue guidance for systemic toxicity. Serum triglycerides, amylase and visual inspection for lipaemia were monitored; amylase remained normal (82–92 U/L) and no pancreatitis, hypertriglyceridaemia requiring intervention, laboratory interference or pulmonary complication was observed. Methylene blue 140 mg (1.0 mg/kg actual body weight) was administered at 16:05 on the basis of published dosing for vasoplegia refractory to catecholamines [5]. Glucose-6-phosphate dehydrogenase status was not known and could not be obtained within the clinical timeframe; no haemolysis occurred, with haemoglobin falling gradually from 14.3 to 12.7 g/dL in a pattern consistent with haemodilution following 6.9 L of fluid resuscitation. The patient had been free of antidepressant therapy for approximately four years, making clinically significant serotonergic interaction with the monoamine oxidase-inhibiting properties of methylene blue unlikely. Transient interference with pulse oximetry is recognised following methylene blue administration; no fall in oxygen saturation was documented, and contemporaneous nursing records noted vital signs to be unchanged.

The arterial blood gas at 17:00 showed a lactate of 6.1 mmol/L, a fall of 42% from the preceding value, with the noradrenaline infusion rate unchanged across that interval (Table 1; Fig. 1). HIET had by then been running for approximately three hours, and ongoing vasopressor support, electrolyte replacement, fluid resuscitation and natural drug redistribution and elimination were all continuing concurrently. VA-ECMO was discussed but not initiated. Lactate fell further to 4.8 mmol/L at 18:50 and 3.7 mmol/L at 20:00, when a mean arterial pressure above 65 mmHg was achieved for the first time. All vasopressors were discontinued by 06:00 on day 2.

HIET was accompanied by three concurrent metabolic complications at 16:50: glucose 1.7 mmol/L, potassium 1.7 mmol/L and phosphate 0.27 mmol/L. Oxygen saturation of 89–90% constrained the volume of 10% dextrose that could be administered, and 50% dextrose at 40 mL/h was substituted. Potassium was replaced with four sequential 40 mmol infusions (at least 160 mmol over 24 h), and potassium phosphate with magnesium sulphate 2 g was given at 20:00. A self-terminating run of ventricular tachycardia (approximately 20 beats at 180 beats/min) occurred at 12:35 on day 2 and was treated with 1 g intravenous magnesium sulphate without recurrence.

Creatinine rose from an admission value of 118 µmol/L to a peak of 303 µmol/L on day 3, representing 2.57 times the admission value and meeting KDIGO stage 2 criteria by creatinine (Table 2). Urine output over the first 24 h was approximately 1330 mL against an input of 6875 mL (approximately 0.43 mL/kg/h using actual body weight, or 0.53 mL/kg/h using the adjusted body weight of 110 kg); the patient remained non-oliguric and urine output criteria did not exceed stage 2 at any point. It is noted that the admission creatinine of 118 µmol/L was itself above the patient’s documented pre-admission baseline of 87 µmol/L recorded in 2024, against which the peak would represent 3.48 times baseline; the more conservative admission-based classification of stage 2 has been applied throughout. No renal replacement therapy was required. Renal function improved steadily and the patient was discharged on day 9 with a creatinine of 165 µmol/L. Outpatient monitoring showed complete recovery, with a creatinine of 103 µmol/L at eight days after discharge and 81 µmol/L with an eGFR of 87.5 mL/min/1.73 m² at 3.5 months, below the admission value and consistent with the long-term baseline (Table 2).

**Table 2:** Serial renal function from admission to outpatient follow-up at 3.5 months, with KDIGO staging by creatinine criterion referenced to the admission value of 118 µmol/L. Urine output criteria did not exceed stage 2 at any point and no renal replacement therapy was required. eGFR, estimated glomerular filtration rate; KDIGO, Kidney Disease: Improving Global Outcomes.

**Date / time**	**Creatinine (µmol/L)**	**eGFR (mL/min/ 1.73 m²)**	**Urea (mmol/L)**	**Ratio to admission**	**KDIGO stage (creatinine criterion)**
25/05 08:20	118	55.6	5.7	—	Admission reference value
25/05 15:10	139	45.6	6.7	1.18	Below stage 1 threshold
25/05 21:30	169	36.0	Not available	1.43	Stage 1 (rise ≥26.5 µmol/L)
26/05 (early)	200	29.4	7.3	1.69	Stage 1
26/05	222	25.9	7.6	1.88	Stage 1
26/05 14:00	251	22.3	8.2	2.13	Stage 2
27/05	303	17.8	13.7	2.57	Stage 2 (peak)
28/05	283	19.3	16.3	2.40	Stage 2
29/05	254	22.0	16.3	2.15	Stage 2
30/05	230	24.8	16.3	1.95	Stage 1
31/05 10:57	232	24.6	15.8	1.97	Stage 1
01/06	190	31.3	13.0	1.61	Stage 1
02/06 (discharge)	165	37.1	9.8	1.40	Stage 1
10/06 (outpatient)	103	65.5	6.2	0.87	Resolved
07/09 (3.5 months)	81	87.5	6.3	0.69	Full recovery

## 3 Discussion

Three aspects of this case merit discussion.

First, the temporal relationship between administration of ILE and methylene blue and the subsequent metabolic improvement is notable but cannot establish causation. A 42% fall in serum lactate within a single measurement interval, with the noradrenaline dose unchanged, is a striking observation. However, HIET had been administered approximately three hours previously and its haemodynamic effects are recognised to be delayed; vasopressor support, calcium, fluid resuscitation and electrolyte correction were continuing; and natural redistribution and elimination of the absorbed drug would be expected to begin within this timeframe. ILE and methylene blue were given 35 min apart, so their individual contributions cannot be separated. The mechanisms by which each might act are plausible: ILE is proposed to act through lipid sequestration, with the high lipophilicity of amlodipine (logP approximately 3.0) favouring partition into the infused lipid phase and reducing free drug available at L-type calcium channels [4], [6]; methylene blue inhibits soluble guanylate cyclase, limiting nitric oxide-mediated cyclic GMP accumulation and restoring vascular smooth muscle tone through a pathway distinct from adrenergic and vasopressin receptor signalling [5], [7]. Studies in human septic shock suggests effects extending beyond guanylate cyclase inhibition, including normalisation of nitric oxide end-products and restoration of vasopressor responsiveness [8], [9]. Whether comparable mechanisms operate in CCB-induced vasodilatory shock is unknown. Aggarwal et al. previously reported haemodynamic improvement following methylene blue in combined beta-blocker and CCB overdose, without concurrent ILE [10]. The case reported by Mengistie et al. provides useful context: a patient who ingested a comparable quantity of amlodipine with an NSAID and who recovered following calcium, vasopressors and HIET without ILE or methylene blue, illustrating that multimodal conventional therapy alone can be sufficient and reinforcing the limits of attributing recovery in our patient to any single intervention [3].

Second, three concurrent metabolic complications of HIET — glucose, potassium and phosphate nadirs occurring simultaneously — are, to our knowledge, not previously described together. Hypokalaemia was present before HIET was commenced (potassium 2.5 mmol/L at presentation, reflecting indapamide-induced kaliuresis) and was compounded by insulin-driven intracellular shift. The phosphate nadir of 0.27 mmol/L reflects a similar mechanism superimposed on diuretic-induced urinary loss. Severe hypophosphataemia impairs diaphragmatic contractility and mitochondrial adenosine triphosphate synthesis and is relevant to ventilatory weaning, yet is not consistently addressed in published HIET protocols [11]. The constraint imposed by borderline oxygen saturation, which precluded adequate volumes of 10% dextrose and required substitution with 50% dextrose, is a practical consideration deserving wider recognition. These observations support one- to two-hourly monitoring of glucose, potassium and phosphate during HIET, with defined replacement targets and concentrated dextrose immediately available.

Third, the renal injury was multifactorial. Contributing factors included systemic hypotension, NSAID-mediated inhibition of afferent arteriolar vasodilation, ACE inhibitor-mediated loss of efferent arteriolar tone, diuretic-induced volume depletion and the patient’s pre-existing impairment [2]. Creatinine continued to rise for approximately 36 h after vasopressors were discontinued. This pattern is consistent with established tubular injury rather than ongoing hypoperfusion, although urinary biomarkers, microscopy and histology were not obtained and the mechanism cannot be confirmed. The complete recovery of renal function demonstrated at 3.5 months, with creatinine returning below the admission value, indicates that the injury was fully reversible without renal replacement therapy.

Limitations of this report are substantial and should be emphasised. Quantitative plasma concentrations of the ingested agents were unavailable, so the diagnosis rests on history and a compatible clinical pattern rather than analytical confirmation. Blood ethanol was not measured. Echocardiography was not performed, so the relative contributions of vasodilation and myocardial depression to the shock state cannot be determined. The individual contributions of ILE, methylene blue and HIET to the observed recovery cannot be separated. G6PD status was not determined before methylene blue administration, and the identity of the previously discontinued antidepressant was not documented. This is a single uncontrolled observation and no causal inference is possible.

## 4 Patient perspective

The patient was informed of the severity of his presentation during his admission. He stated that he had not appreciated the potential lethality of the quantity ingested and described the act as impulsive. He engaged with the mental health liaison team and, with written consent, asked that the case be reported if it might benefit others.

## 5 Conclusion

Sequential administration of intravenous lipid emulsion and methylene blue was temporally associated with rapid metabolic and haemodynamic recovery in a patient with refractory circulatory shock following polypharmacy antihypertensive and NSAID overdose, in whom seven hours of conventional treatment had not achieved haemodynamic targets. The individual contribution of these agents cannot be established from a single case, and concurrent therapies, particularly HIET, may have contributed. These agents may represent rescue options when standard therapy is insufficient, and this experience indicates that such an approach is feasible in a regional hospital without on-site ECMO. The concurrent glucose, potassium and phosphate nadirs observed during HIET warrant specific attention in monitoring protocols. Prospective data and larger case series are required.

## CRediT authorship contribution statement

**Syed Hussain Danial:** Writing – original draft. **David Michael Honan:** Writing – review & editing.

## Ethical approval and consent to publish

All procedures followed were in accordance with the ethical standards of the responsible committee on human experimentation (institutional and national) and with the Helsinki Declaration of 1975, as revised in 2000. Written informed consent was obtained from the patient for publication. The signed consent form is retained by the corresponding author.

## Patient consent

Written informed consent was obtained from the patient for publication of this case report. Additional consent was obtained as the patient is identifiable within this report. The signed consent form is retained by the corresponding author and will be provided on request.

## CARE checklist

Prepared in accordance with CARE guidelines; completed checklist provided as a supplementary file.

## Funding

This research did not receive any specific grant from funding agencies in the public, commercial, or not-for-profit sectors.

## Declaration of Competing Interest

The authors declare that they have no known competing financial interests or personal relationships that could have appeared to influence the work reported in this paper.

## Data Availability

No data was used for the research described in the article.
