# Supplementary material for: Refractory circulatory shock following intentional polypharmacy antihypertensive and NSAID overdose: Clinical recovery temporally associated with sequential intravenous lipid emulsion and methylene blue — A case report
Source: Toxicol Rep. 2026 Nov 6;17:102371. doi: 10.1016/j.toxrep.2026.102371 (PMC13769549; doi:10.1016/j.toxrep.2026.102371)
Supplement: Supplementary file 1 [file mmc1.docx]

**CARE Checklist of Information to Include When Writing a Case Report**

**Manuscript: TOXREP 102371 (TOXREP-D-26-00679R1)**

Title: Refractory Circulatory Shock Following Intentional Polypharmacy Antihypertensive and NSAID Overdose: Clinical Recovery Temporally Associated with Sequential Intravenous Lipid Emulsion and Methylene Blue — A Case Report

Authors: Syed Hussain Danial; David Michael Honan

Journal: Toxicology Reports

*All thirteen items of the CARE checklist are addressed in the manuscript. Section numbers refer to the numbered sections of the submitted manuscript; page numbers are not given as pagination will change at typesetting.*

| **Item** | **Checklist item** | **Description** | **Reported in** |
| --- | --- | --- | --- |
| **1** | Title | The words “case report” appear in the title, with the area of focus | Title page — title ends “— A Case Report” |
| **2** | Key words | Two to five key words identifying the diagnoses and interventions | Abstract, following Conclusion — six key words provided |
| **3a** | Abstract: Introduction | What is unique and why it matters | Abstract, Background |
| **3b** | Abstract: Case presentation | Main symptoms, clinical findings, diagnoses, interventions, outcomes | Abstract, Case report |
| **3c** | Abstract: Conclusion | Main take-away lessons | Abstract, Conclusion |
| **4** | Introduction | Brief background summary with references | Section 1, Introduction, with references [1–5] |
| **5a** | Patient information | De-identified demographic and other patient information | Section 2, paragraph 1 — 64-year-old man, 135 kg |
| **5b** | Patient information | Main concerns and symptoms of the patient | Section 2, paragraphs 1 and 3 |
| **5c** | Patient information | Medical, family and psychosocial history, including diet, lifestyle and genetic information where relevant; relevant comorbidities and past interventions with outcomes | Section 2, paragraph 1 — treated hypertension on Coverdine; history of depression with antidepressant therapy discontinued approximately four years earlier; recent bereavement; ethanol co-ingestion |
| **6** | Clinical findings | Describing the relevant physical examination and other clinical findings | Section 2, paragraph 3 — prehospital and Emergency Department observations, pulse pressure, temperature |
| **7** | Timeline | Relevant data from the episode of care organised as a timeline | Table 1 — serial arterial blood gases and clinical events with times; Table 2 — serial renal function to 3.5 months; Figure 1 — three-panel temporal plot |
| **8a** | Diagnostic assessment | Diagnostic testing, including physical examination, laboratory testing and imaging | Section 2, paragraphs 2 and 3; Tables 1 and 2 |
| **8b** | Diagnostic assessment | Diagnostic challenges | Section 2, paragraph 2 — quantitative plasma drug concentrations unavailable; screen does not detect the ingested agents; blood ethanol not measured. Section 2, paragraph 3 — echocardiography not performed |
| **8c** | Diagnostic assessment | Diagnostic reasoning, including other diagnoses considered | Section 2, paragraph 2 — exclusion of paracetamol, opiates, benzodiazepines, cannabis, tricyclic antidepressants, cocaine, sepsis and thyroid dysfunction; clinical pattern described as compatible with, not diagnostic of, CCB toxicity |
| **8d** | Diagnostic assessment | Prognostic characteristics where applicable | Section 1 — reported mortality of CCB overdose without advanced organ support |
| **9a** | Therapeutic intervention | Types of intervention, pharmacological and other | Section 2, paragraphs 4 and 5 — fluids, noradrenaline, vasopressin, calcium gluconate, HIET, intravenous lipid emulsion, methylene blue, electrolyte replacement, magnesium sulphate |
| **9b** | Therapeutic intervention | Administration of intervention, including dose, strength, duration and frequency | Section 2, paragraphs 4 and 5; Table 1. Doses expressed in mg/kg for methylene blue and mL/kg and g/kg for lipid emulsion, with infusion durations |
| **9c** | Therapeutic intervention | Changes in intervention, with rationale | Section 2, paragraph 6 — substitution of 50% dextrose for 10% dextrose owing to oxygen saturation constraints on fluid volume |
| **10a** | Follow-up and outcomes | Clinician and patient-assessed outcomes where appropriate | Section 2, paragraphs 6 and 7; Section 4, Patient perspective |
| **10b** | Follow-up and outcomes | Important follow-up diagnostic and other test results | Table 2 — renal function to outpatient follow-up at 3.5 months, demonstrating complete recovery |
| **10c** | Follow-up and outcomes | Intervention adherence and tolerability, and how this was assessed | Section 2, paragraph 5 — monitoring of triglycerides, amylase and lipaemia; haemoglobin trend excluding haemolysis; oxygen saturation unchanged after methylene blue |
| **10d** | Follow-up and outcomes | Adverse and unanticipated events | Section 2, paragraph 6 — concurrent glucose, potassium and phosphate nadirs during HIET; self-terminating ventricular tachycardia on day 2 |
| **11a** | Discussion | Strengths and limitations of the management of this case | Section 3, final paragraph — limitations stated in full |
| **11b** | Discussion | Relevant medical literature with references | Section 3 — references [2–11], including comparison with Mengistie et al. [3] |
| **11c** | Discussion | Rationale for conclusions, including assessment of possible causes | Section 3, paragraph 2 — alternative explanations including delayed HIET effect, concurrent therapies and natural drug elimination; inability to separate individual contributions |
| **11d** | Discussion | Main take-away lessons of this case report | Section 5, Conclusion |
| **12** | Patient perspective | The patient should share their perspective on the treatment they received | Section 4, Patient perspective |
| **13** | Informed consent | Did the patient give informed consent? Please provide if requested | Yes. Title page and Ethical approval and consent to publish section. Written informed consent obtained; signed form retained by the corresponding author and available on request |

Reference: Gagnier JJ, Kienle G, Altman DG, Moher D, Sox H, Riley D; CARE Group. The CARE guidelines: consensus-based clinical case reporting guideline development. BMJ Case Rep. 2013;2013:bcr2013201554.

Completed by: Syed Hussain Danial MBBS MCAI FCAI, corresponding author

Date: 4 November 2026
